# Synthesis and Optimization of Ni-Based Nano Metal–Organic Frameworks as a Superior Electrode Material for Supercapacitor

**DOI:** 10.3390/nano14040353

**Published:** 2024-02-13

**Authors:** Carolina Manquian, Alberto Navarrete, Leonardo Vivas, Loreto Troncoso, Dinesh Pratap Singh

**Affiliations:** 1 Department of Metallurgical Engineering, Faculty of Engineering, University of Santiago of Chile (USACH), Av. Lib. Bernardo O’Higgins 3363, Estación Central, Santiago 9170022, Chile; carolinamanquian@gmail.com (C.M.); albertonavarretev@gmail.com (A.N.); 2 Physics Department, Millennium Institute for Research in Optics (MIRO), Faculty of Science, University of Santiago of Chile (USACH), Avenida Victor Jara 3493, Estación Central, Santiago 9170124, Chile; vivasleonard@gmail.com; 3 Department of Electrical Engineering, Universidad Técnica Federico Santa Maria, Santiago 8940000, Chile; 4 Institute of Mechanical Engineering, MIGA Millennium Institute, University Austral of Chile, Valdivia 5090000, Chile; loreto.troncoso@uach.cl

**Keywords:** nanomaterials, Ni-based, metal–organic frameworks, H_2_bdt, electrochemical characterization, supercapacitors

## Abstract

Metal–organic frameworks (MOFs) are hybrid materials that are being explored as active electrode materials in energy storage devices, such as rechargeable batteries and supercapacitors (SCs), due to their high surface area, controllable chemical composition, and periodic ordering. However, the facile and controlled synthesis of a pure MOF phase without impurities or without going through a complicated purification process (that also reduces the yield) are challenges that must be resolved for their potential industrial applications. Moreover, various oxide formations of the Ni during Ni-MOF synthesis also represent an issue that affects the purity and performance. To resolve these issues, we report the controlled synthesis of nickel-based metal–organic frameworks (NiMOFs) by optimizing different growth parameters during hydrothermal synthesis and by utilizing nickel chloride as metal salt and H_2_bdt as the organic ligand, in a ratio of 1:1 at 150 °C. Furthermore, the synthesis was optimized by introducing a magnetic stirring stage, and the reaction temperature varied across 100, 150, and 200 °C to achieve the optimized growth of the NiMOFs crystal. The rarely used H_2_bdt ligand for Ni-MOF synthesis and the introduction of the ultrasonication stage before putting it in the furnace led to the formation of a pure phase without impurities and oxide formation. The synthesized materials were further characterized by powder X-ray diffraction (XRD) technique, scanning electron microscopy (SEM), and UV–vis spectroscopy. The SEM images exhibited the formation of nano NiMOFs having a rectangular prism shape. The average size was 126.25 nm, 176.0 nm, and 268.4 nm for the samples (1:1)s synthesized at 100 °C, 150 °C, and 200 °C, respectively. The electrochemical performances were examined in a three-electrode configuration, in a wide potential window from −0.4 V to 0.55 V, and an electrolyte concentration of 2M KOH was maintained for each measurement. The charge–discharge galvanostatic measurement results in specific capacitances of 606.62 F/g, 307.33 F/g, and 287.42 F/g at a current density of 1 A/g for the synthesized materials at 100 °C, 150 °C, and 200 °C, respectively.

## 1. Introduction

Metal–organic frameworks are hybrid materials that consist of a central metal ion coordinated by organic ligands to form one-, two-, and three-dimensional structures. They have attracted attention owing to their exceptional properties, such as large surface areas and adaptive structures [1] ). The organic and inorganic components of MOFs allow the modification of their size, geometry, branching, functional groups and pore size to obtain MOFs with different topologies, functionalized surface areas, and specific properties. Recently, MOFs have been explored in various application areas such as gas absorption and selection [2] catalysis [3], luminescence and sensors [4], biomedicine [5] imaging and drug release [6] optics [7] photoelectrochemistry [8], and electrochemistry [9], among others. They are active elements in energy- and power-related devices, including rechargeable batteries, supercapacitors (SC), and SC-hybrid batteries (BSH) [10] mainly because of their high specific surface area, controllable chemical composition, and adjustable pore size and shape, as well as their diverse functionalization, periodic ordering, and abundant active sites. Pure phase formation, free from ligands and oxides, unreacted salt impurities, low yield, etc. are the challenges that need to be resolved for various applications and commercialization. Moreover, due to their favorable porosity, MOFs are limited in their low electrical conductivity and chemical stability [9,11,12] ). To overcome these limitations, different synthesis methods have been proposed and studied to improve the pure phase synthesis and conductivity of MOFs for their application as SCs. The synthesis of MOF-derived metal oxides, or generating composites, mixing the MOF with an active conductive material, such as carbon family, metal oxides, metal nanoparticles, or polymer has been proposed [12,13]); however, these methods use the MOF as a sacrificial material, destroying the crystalline structure and porosity of the MOF. In order to obtain synergy between both components, time and resources may be wasted [14]. Another method is that of dimensional reduction, or the formation of zero-, one-, and two-dimensional nanomaterials which enhances the surface area and conductivity of the MOF without losing its intrinsic properties. It has been shown that nanoparticles of MOFs (nano MOFs) have increased in electrical conductivity, showing good performance when applied as electrode materials in the supercapacitors [15] (however, the synthesis of nanometric NiMOFs has been limited to the use of compact ligands, [16,17,18]. Ouellette et al. [19] synthesized the Ni(II) [$Ni_{2}{\left(H_{0.67}bdt\right)}_{3}]\cdot 10.5H_{2}O$] phase structure, a three-dimensional open structure, where each Ni(II) site adopts distorted octahedral coordination within the chain by binding to the nitrogen donors in six bdt ligands, where the ligand used is $1,4-bis(1H-tetrazol-5-yl)benzene(H_{2}bdt$). The ligand H_2_bdt has four nitrogen donor atoms per heteroaromatic ring, and studies have shown that the ligand $H_{2}bdt$ can participate in up to five different types of coordination modes with metal ions in the construction of organometallic structures [20]. Therefore, it is a potential ligand, although it is sparsely used for NiMOFs to create new coordinated frameworks that can go from 1D to 3D materials.

In this work, using the hydrothermal method, nickel-based MOFs were synthesized, using nickel chloride and H_2_bdt as ligands. The effective parameters of the synthesis were optimized to achieve pure nano NiMOF such as the introduction of a magnetic stirring stage and synthesis temperature, etc. This resulted in the formation of a large number of nano NiMOFs with homogeneous rod-like structures. The samples were microstructurally and structurally characterized using powder X-ray diffraction (pXRD), scanning electron microscopy (SEM), FTIR, and UV–visible spectroscopy. In addition, the different synthesized samples were dielectrically and electrochemically characterized, obtaining that, when using a nano NiMOF, with a longer ligand, the potential window is wider than those reported, obtaining an improved specific capacitance, when magnetic stirring is used and the hydrothermal synthesis temperature is of 100 °C, using three-electrode configurations in an alkaline medium of 2MKOH.

## 2. Materials and Methods

### 2.1. Materials

All the synthesis chemicals and reactants such as 1,4-dicyanobenzene $(C_{6}H_{4}{\left(CN\right)}_{2}$) (Sigma-Aldrich, Burlington, Massachusetts, MA, USA), sodium azide ($NaN_{3}$) (Sigma-Aldrich), zinc chloride (NaCl), nitric acid, 70% ACS ($HNO_{3}$) (Fermont), nickel (II) chloride hexahydrate ($NiCl_{2}\cdot 6H_{2}O$), tetrabutylammonium hydroxide solution (HT), technical, ~40% in $H_{2}O$ (~1.5 M) (Sigma-Aldrich), ethanol, etc. and reagents for electrochemical analysis such as poly(vinylidene fluoride) (PVDF) (Sigma-Aldrich), 1-methyl-2-pyrrolidine (NMP) (Sigma-Aldrich), potassium hydroxide (KOH) (EMSURE^®^, Darmstadt, Germany), activated charcoal (AC) (Supelco Analytical, St. Louis, SL, United States.), and conductive silver printing ink (Sigma-Aldrich) were purchased from different companies and utilized, without any further purifications.

### 2.2. Synthesis Methods

#### 2.2.1. Synthesis of Ligand $H_{2}bdt$

Ligand synthesis was carried out using the modified synthesis [21]. First, various chemicals and reagents such as (4 mmol, 260.28 mg) sodium nitrate, (1 mmol, 136.30 mg) zinc chloride, (2 mmol, 256.26 mg) 1,4-dicyanobenzene, and (8 mL) $H_{2}O$ were mixed together. Furthermore, ~2.5 mL of $HNO_{3}$ was slowly added and the pH was adjusted between 2 and 3. The mixture was heated at 120° C for 48 h. After 48 h, the mixture was hot-filtered and washed with ethanol to obtain yellow color crystals. The material was stored in a vacuum desiccator at room temperature for further utilization.

#### 2.2.2. Synthesis of NiMOF

**Figure 1:**
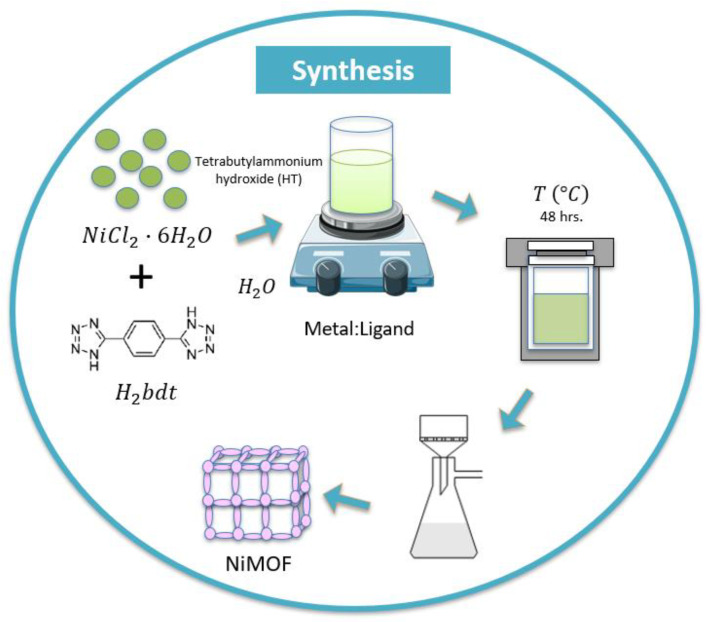
Experimental methods for hydrothermal synthesis of NiMOF.

For the hydrothermal synthesis, the following method of modified synthesis reported by Ouellette et al. [19] was followed as described in Figure 1.

The hydrothermal synthesis of the sample at a molar concentration metal-to-ligand ratio of 1:1 was carried out in a 125 mL Teflon autoclave. First, for the molar ratio of 1:1, we used 0.555 g (2.33 mmol) of $NiCl_{2}\cdot 6H_{2}O$, 0.500 g (2.33 mmol) of $H_{2}bdt$ as the ligands were further dissolved in 102 mL (5657 mmol) of distilled water by adding a 2 mL of Tetrabutylammonium solution. The mixture was transferred into a stainless-steel autoclave, tightly closed, and put in the furnace at 150 °C for 48 h. After the reaction time was completed, the autoclave was automatically cooled down to room temperature and the solution was further filtered and washed with ethanol to remove the by-products. The resulting material was dried in a vacuum at room temperature.

As a synthesis modification and introduction of the magnetic stirring stage, first, the mixture was prepared using the same previous proportions and after combining the metal salt, the ligand, and the distilled water, the resulting solution was left for magnetic stirring for 2 h and 20 min at room temperature. Then, 2 mL of Tetrabutylammonium solution was added and left stirring for 10 min more. The mixture was transferred into an autoclave and put in the furnace at 150 °C for 48 h, as described earlier. When the autoclave had cooled down to room temperature, the mixture was filtered, washed with ethanol, and stored under vacuum. This sample with the modified method is named (1:1)s.

Finally, using the same procedure as mentioned above, the synthesis temperature was changed to 100 °C and 200 °C, and the reaction time was kept constant at 48 h.

### 2.3. Structural, Microstructural, and Spectral Characterization

The X-ray diffraction analysis was carried out in the D2 PHASE equipment, Bruker brand (Billerica, Massachusetts, MA, United States), with CuKα radiation (1.5418 Å). Diffractions were recorded in an angle 2 theta range from 5° to 40°, with a step size of 0.04 to 0.5 s/step. The low- and high-magnification images were taken by scanning electron microscope (SEM) of Zeiss EVO Ma10 (Oberkochen, Germany ) with an accelerating voltage of 20 KeV. Fourier transform infrared spectrometer, Bruker IFS66V (Billerica, MA, USA) in a wavelength range of 450 nm to 4000 nm. UV–visible absorption spectra were recorded by Perkin Elmer, Lambda 750 S spectrometer (PerkinElmer, Inc., Waltham, MA, USA) in a wavelength range of 200 nm to 800 nm. The thermogravimetric analysis (TGA) was performed on the Perkin Elmer Thermogravimetric Analyzer TGA 4000 equipment (Shelton, Connecticut, CT, USA), controlled by the Pyris Series TGA 4000 software of 2.2 bar in the $N_{2}$ atmosphere. The dielectric spectroscopy was performed on IM 3570 Impedance Analyzer, for a frequency range of 5 Hz–5 MHz, a level of 1.0 V, and an average of 10 points. The contact diameter used was 6.4 mm, with an initial contact resistance of 0.3 ohm. The samples were tablets pressed at 3 tons.

### 2.4. Electrochemical Characterization

#### 2.4.1. Electrode Preparation

The working electrode was prepared by forming a paste of active material (NiMOF), commercial grade activated charcoal (AC), poly(vinylidene fluoride) (PVDF) as a binder, and 1-methyl-2-pyrrolidone as a solvent, by maintaining a proportion of active material:AC:binder = 6:3:1, respectively. A higher proportion of AC is utilized as the active material, being a MOF, is a poor conductor; however, this proportion is also reported in various articles [22,23]. Nevertheless, it also refers to the electrode material containing a lower percentage of active material which can increase the performance if a suitable conductor is utilized with the optimum ratio, or the composites of MOFs are formed with some conducting materials. To obtain the mixture, 60 mg of NiMOF, 30 mg of AC, 10 mg of PVDF, and 1 mL of 1-methyl-2-pyrrolidone were stirred for 2 h at room temperature; then, after left stirring at 60 °C for 1 h, a paste was obtained. The as-obtained paste material was spread on a Ni-Foam plate of 1 cm^2^ and left on a hot plate at 100 °C until the solvent evaporated. The electrical contacts were made of a thin copper wire glued with a conductive silver paint.

#### 2.4.2. The Standard Three-Electrode Configuration

For the electrochemical measurements, the standard three-electrode configuration was utilized by using an Ag/AgCl electrode as a reference electrode (RE), a platinum wire as the counter electrode (CE), the synthesized materials as a working electrode (WE), and an alkaline solution of 2M KOH as an electrolyte. The measurements were made by using the Metrohm Autolab instrument, through the Intello 1.3 program and Interface 5000 E Gamry Instrument (Gamry Instruments, 734 Louis Drive, Warminster, PA 18974, United States of America), at room temperature. The electrochemical properties and capacitance measurements of the electrodes were studied by cyclic voltammetry (CV), galvanostatic electrochemical impedance spectroscopy (EIS), and charge–discharge (CD) over a potential window of [−0.40 to 0.55] V. For the cyclic voltammetry, different sweep rates such as $5\;mVs^{-1}$, $10\;mVs^{-1}$, $15\;mVs^{-1}$, $20\;mVs^{-1}$, $25\;mVs^{-1}$, $50\;mVs^{-1}$, $100\;mVs^{-1}$, $150\;mVs^{-1}$, $200\;mVs^{-1}$, $250\;mVs^{-1}\;were\;utilized$. Impedance spectroscopy measurements were carried out in the frequency range of 0.1 MHz–0.1 Hz. While the charge–discharge measurements were made in a potential window of [−0.40–0.55] V for currents of 1 $Ag^{-1}$, 2 $Ag^{-1}$, 3 $Ag^{-1}$, 4 $Ag^{-1}$, 5 $Ag^{-1}$, and 10 $Ag^{-1}$.

In the three-electrode configuration, the specific capacitance can be calculated by cyclic voltammetry curves according to the following equations:

(1) $$C_{s}=\frac{\int I\;dV\;}{\upsilon \;m\;\Delta V},$$

where $C_{s}$ ($Fg^{-1}$) is the specific capacitance of the closed cyclic voltammetry (CV) curve, *I* (A) is the current related to voltage V, Δ*V* (V) is the voltage window, υ ($mVs^{-1}$) is the scan rate, and *m* (g) is the mass of the active material.

Additionally, the specific capacitance can be calculated by galvanostatic charge–discharge (GCD), so for battery-type materials, GCD data are calculated by using the equation:

(2) $$C_{CD}=\frac{I\times ∆t}{∆V\times m},$$

where $C_{CD}$ is charging–discharging capacitance, Δ*V* denotes the operational voltage window (V), *I* correspond to the discharge current (A), and *m* is the mass of active materials (g) [24].

## 3. Results and Discussion

### 3.1. Characterization of Ligand

**Figure 2:**
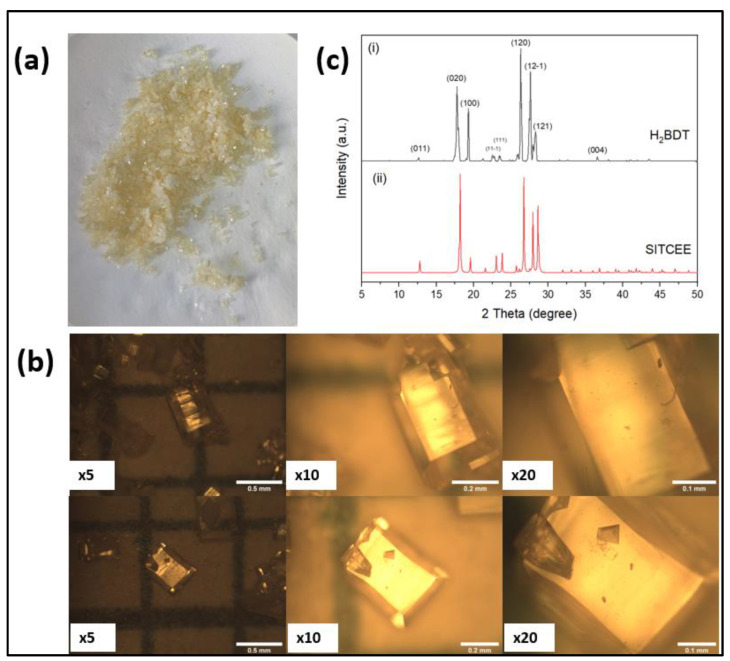
(**a**) Optical images of the ligand with the naked eye; (**b**) optical microscopic images using magnitudes of ×5, ×10, and ×20, respectively; and (**c**) powder X-ray diffraction pattern of the synthesized ligand by modified method (CCDC reference no. 674390).

Figure 2a shows the optical image of the as-synthesized ligand via hydrothermal synthesis. The synthesized ligand was in powder form and visibly light-yellow in color. Figure 2b shows the images recorded by an optical microscope at different magnifications. The images show well-defined micron-sized crystals having different dimensions. The powder X-ray diffraction (Figure 2c) confirms that the synthesized ligand has all the peaks matched with SITCEE. CCDC reference no. 674390 as previously reported by He et al. [21]. The crystals have a preferential growth in the crystallographic plane of (120) which confirms the formation of the reported phase using the modified synthesis method of the ligand.

### 3.2. Structural Microstructural Characterization of Synthesized NiMOFs

By utilizing the method as described in the experimental sections, various NiMOFs are synthesized and characterized for the structural, microstructural, and pure phase formation of the products. Additionally, effective parameters were optimized to have pure phase and nano MOFs.

**Figure 3:**
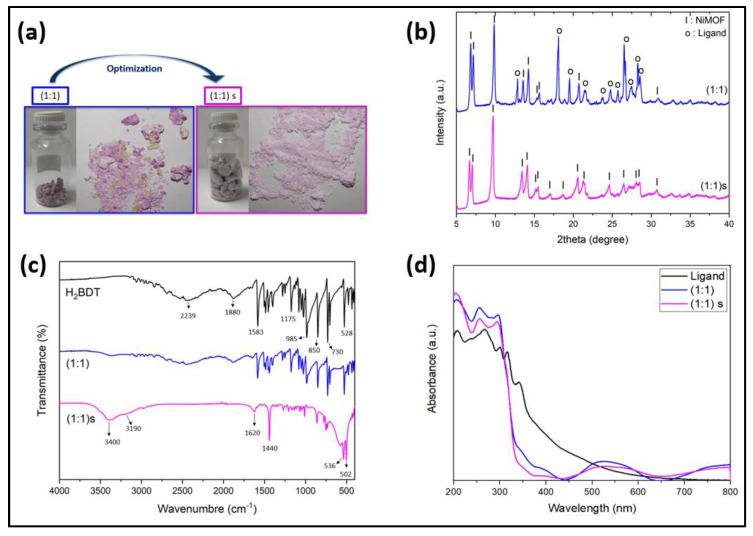
(**a**) Optical images of the NiMOF synthesized at ratios (1:1) and with stirring (1:1)s; (**b**) powder X-ray diffractogram of the NiMOFs (CCDC 826920) samples (1:1) and (1:1)s; (**c**) FT-IR spectrum for the samples (1:1), (1:1)s, and ligand; (**d**) UV–visible absorption spectrum for the samples (1:1) and (1:1)s compared with the ligand.

Figure 3a shows the optical images of the synthetized samples. For the sample (1:1), it is observed that they are in the form of pink and/or purple powder with some yellow crystal formation. These yellow-colored crystals, which were the most observed, may contain excess ligand residues that could not be utilized during the hydrothermal synthesis. In fact, in the hydrothermal synthesis, a green color powder material also formed as a by-product, which corresponds to nickel hydroxide ${\beta -Ni(OH)}_{2}$. When this stirring was added to the hydrothermal synthesis, (1:1)s, it was observed that a more homogeneous, light pink color powder was obtained, which was free from the larger-sized yellow color by-product crystal, in higher volume and yield in comparison to the samples wherein the stirring stage is not involved. The yield obtained for the sample (1:1) was less than 27%, considering that the ligand and nickel oxide crystals were obtained, while the yield of the sample (1:1)s was 37%. Therefore, stirring not only improved the purity but also improved the yield.

X-ray analysis of the synthesized products obtained at (1:1) and (1:1)s is shown in Figure 3b. The pXRD pattern of the sample (1:1), indicates the formation of mixed phases which were indexed with the peaks of the ligand and NiMOF (CCDC 826920), as reported by Ouellette et al. [19]. The most intense peaks correspond to the structure of NiMOF, situated at angles of 2θ: 9.8°, 6.8°, and 7.2°, with preferential crystallographic growth directions corresponding to the (021), (020), and (001) planes, respectively. In the diffractogram, the characteristic peaks of the ligand were also observed, presenting higher intensity peaks at angles of 18.1° and 26.5°, which correspond to the (002) and (120) crystallographic planes, respectively. When using magnetic stirring, we observed homogeneous phase formation corresponding to that of NiMOF reported by Ouellette et al. [19]. For the sample (1:1)s, the most intense peaks corresponded to the angles of 2θ: 6.7°, 7.0°, 9.7°, 13.4°, and 14.1°, where the preferential direction was clearly observed for the (021) plane.

The FTIR spectra of the ligand, (1:1), and (1:1)s are shown in Figure 3c. For the ligand, the peak at 2239 cm^−1^ is assigned to the C≡N bond. A bending vibration of 1583 cm^−1^ is attributed to the asymmetric stretching vibration of the –COO– carboxyl group of the ligand, and, in the range of 1500–1400 cm^−1^, it is depicted as the stretching vibration of C–C in the ring of ligand, while the peaks at 1175 and 985 cm^−1^ are ascribed to C–N stretching vibration, corresponding to the aliphatic amide of ligand. The peak between 850 and 730 cm^−1^ results from the absorption band from vibrations of the C–H groups, while the peak at 528 cm^−1^ and a lesser wavenumber may be attributed to the amide group [25]. For the sample (1:1), the vibration peaks are similar to the ligand but with less intense peaks, showing a vibration peak around 3400 cm^−1^. For the sample (1:1)s, the vibrational peaks at 3400 and 3190 cm^−1^ are due to the O–H stretching vibration of water molecules, which suggests that water molecules are present within the NiMOF structure. For the range between 3000 and 2850 cm^−1^, show the bands depicted as the stretching vibration present in the C–H group. The peak at 1620 cm^−1^ is ascribed to the asymmetric stretching vibration of the coordinated (–COOC–) carboxylic group of ligands, confirming that the ligand is well coordinated with the metal ions. The band located at 1440 cm^−1^ is assigned to the C–N bond attributed to the –COO– ligand [26]. However, the peaks at 536 and 502 cm^−1^ are assigned to the Ni–O–H bending vibration and Ni–O stretching, respectively [27]. From the figure wherein the nickel salt is added to the synthesis, there is metal–ligand coordination only when stirring is added in to the synthesis; thus, nickel can form metal-to-ligand bonding, where the peaks of the ligand disappear and form a new peak, which confirms the formation of the metal-to-ligand bond.

The UV–vis absorption spectra of the ligand and the samples (1:1) and (1:1)s were recorded in solid form in the range of 200–800 nm at room temperature, as shown in Figure 3c. The image shows that the ligand absorbs the entire UV–visible spectrum and has five characteristic peaks situated at 208 nm, 267 nm, 300 nm, 316 nm, and 341 nm, whereas, in this region, there is the presence of conjugated bond type n-σ* transition and n–π* transition of the bond of C–N, C=O, and N=N [28]. Then, decay is observed where two lumps can be seen, between 380–440 nm and 440–510 nm. In the figure, it can be observed that the spectrum of the two synthesized samples has similar peaks and shapes, absorbing throughout the UV–visible spectrum due to metal-to-ligand change transfer (MLCT). For the sample (1:1), the first two intense peaks for the three samples are in the range of 207 nm–257 nm bands, which would correspond to π–π* transitions. On the other hand, the third band is located at 295 nm for sample (1:1), which is in a band that is characteristic of the nitrate ion complex, corresponding to the n–π* transition. It is also observed that there is a pronounced peak centered around 525 nm, which corresponds to the d–d transition attributed to the Ni^2+^ ion, specifically to spin-allowed transition A2g3→T1g3, which is responsible for the purple color of the sample. Finally, an increase in intensity is observed around 800 nm, and this band is also associated with the d–d transition and corresponds to A2g3→Eg1, which is the spin-forbidden transition of the octahedral Ni^2+^ ion [29,30]. The sample (1:1) is absorbed while decay presents two bands in the wavelength ranges of 333 nm–372 nm and 375 nm–433 nm, similarly to the behavior of the ligand pattern. For sample (1:1)s, the first peak is observed in the band of 205 nm. The second absorption band is located at 256 nm; however, the position of the third band is located at 294 nm. Similarly, an increase in absorbance is observed in the band of 526 nm in the case of (1:1)s, in addition to the increase around 800 nm, whereas for these last two cases, they correspond to the d–d transition of Ni^2+^ ions. Although the spectra of the without-stirring samples are akin to the stirring ones, the difference can be observed in the intensities which are slightly higher in the stirred case, which may be because the stirring samples are pale in color. Furthermore, the intensities of the bumps observed in the ranges of 333 nm–372 nm and 375 nm–433 nm are considerably decreased in stirred samples, indicating that, in these samples, there is a negligible contribution of the residue’s ligand. When calculating the energy band gap by linear fitting from the Tauc plot using a direct transition, it is obtained that, for the sample (1:1), the band gaps are 3.91 eV, while for the stirred sample, the band gaps are 3.89 eV, wherein the transition energy is maintained for the samples (1:1)–(1:1)s.

**Figure 4:**
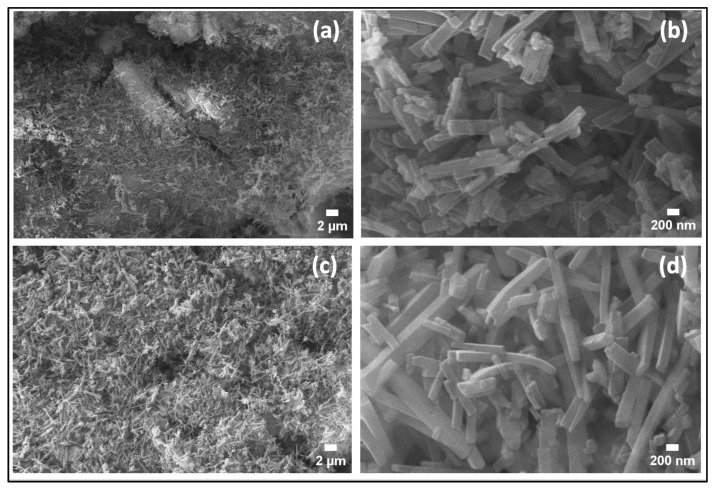
SEM images of (**a**,**b**) the sample (1:1), and (**c**,**d**) for the sample (1:1)s.

The microstructures of the synthesized samples were investigated using SEM. Figure 4a,b show the SEM images of the sample obtained in the case of (1:1), which shows a large particle, with a large number of regular rectangular-shaped rod-like structures on the surface. The panel Figure 4c,d represents the SEM images of the sample (1:1)s, which depict a large number of defined rectangular-shaped rod-like structures. The width of the nanorods varies between 94.9 nm and 292.28 nm, with an average width of 176.0 nm and length ranging from 200 nm to 3 μm. Introducing the magnetic stirring stage before the hydrothermal process has a clear effect on the microstructures of the synthesized samples because of the generation of new nucleation sites forming more homogeneous and thinner nanorods and nanoflake-like structures and 1D structures.

### 3.3. Effect of Temperature

**Figure 5:**
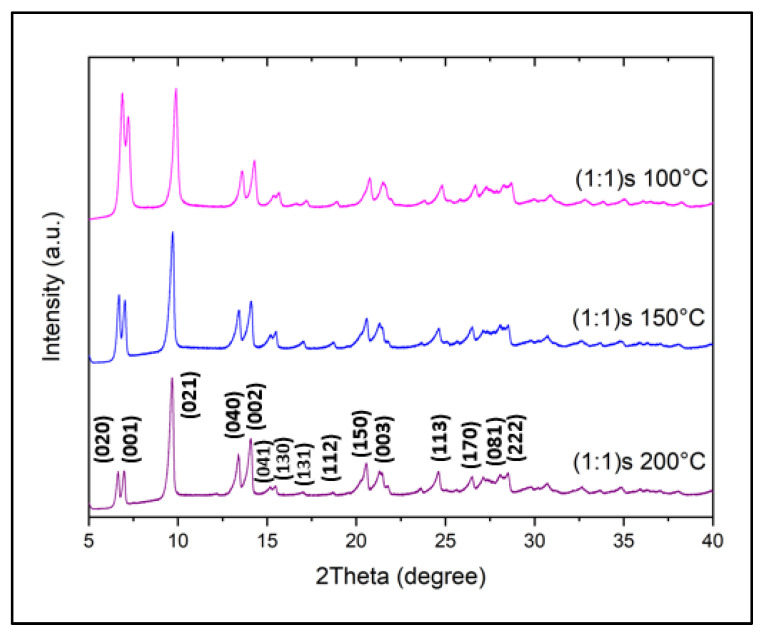
Power X-ray diffraction of the sample (1:1)s at different temperatures of synthesis.

Since we observed in previously obtained results that magnetic stirring improves nanostructures, another parameter of interest for this study is the synthesis temperature in the hydrothermal method, since this not only affects the particle size but also the crystalline structure of the material, and thus, we further analyzed the effect of temperature on the stirred sample. Figure 5 shows the powder XRD pattern of the three samples of with (1:1)s ratios synthesized at different temperatures of 100°, 150, and 200 °C, and named (1:1)s 100 °C, (1:1)s 150 °C, and (1:1)s 200 °C, respectively. It can be seen that all these materials show a similar pattern confirming the formation of NiMOF as reported by Ouellette et al. [19]. For the sample synthesized at 100 °C, (1:1)s 100 °C magenta, union and slight overlapping are observed between the first two consecutive peaks of (020) and (001), where the higher-intensity peak corresponds to the crystallographic plane of (020).

The main differences in the samples synthesized at different temperatures are crystallinity and preferred direction of growth. When the synthesis is carried out at 100 °C, the sample shows softer peaks, where the first two peaks (020) and (001) are as intense as the third peak (021). These two peaks decrease in intensity as the synthesis temperature increases, achieving a lower intensity than the third peak, corresponding to the (021), showing that it is the preferential crystallographic growth direction, namely at 200 °C, which shows their high crystallinity and purity.

**Figure 6:**
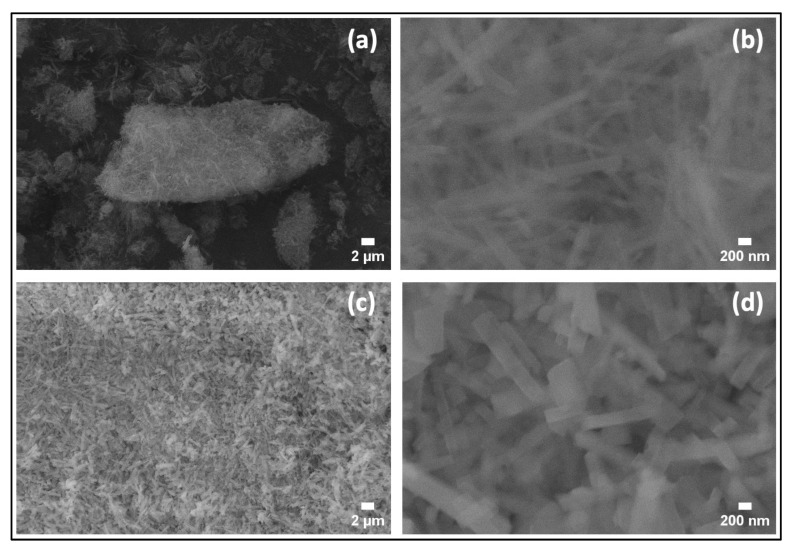
Low- and high-magnification SEM images (**a**,**b**) of the sample (1:1)s 100 °C; and (**c**,**d**) of the sample (1:1)s 200 °C.

The SEM images of the samples (1:1)s synthesized at 100 °C and 200 °C can be seen in Figure 6a,b for the sample (1:1)s 100 °C, and Figure 6c,d for the sample (1:1)s 200 °C). The images exhibited homogeneously distributed rectangular prism-shaped particles with a particle width that varies between 97.4 nm and 155.1 nm for the sample (1:1)s 100 °C, and between 187.0 nm and 349.8 nm for the sample (1:1)s 200 °C. When the synthesis temperature is 100 °C, it is observed that the particles are fine, thin, and elongated; however, particles synthesized at 200 °C resulted in shorter and wider rod-like structures, which implies that the increase in the temperature of the hydrothermal synthesis, in addition to increasing the crystallographic intensity of the (021) plane, also increases the particle size while maintaining the shape of the particle. The SEM images of the sample (1:1)s obtained at 150 °C are already described and discussed in Figure 4c,d.

### 3.4. Results Dielectric Spectroscopy

Table 1 shows the parallel capacitance, parallel resistance, and AC conductivity for the samples synthesized using magnetic stirring at different temperatures at a frequency of 100 Hz.

At 100 Hz, the parallel capacitance is in the order of picofarads, while the parallel resistance is in the order of mega Ohms. In the case of samples with magnetic stirring (1:1)s at different synthesis temperatures, the trend is clear, namely, the capacitance decreases as the synthesis temperature increases, which may be due to an increase in the particle size of the samples.

However, the parallel resistance is at its highest when the sample is synthesized at 200 °C, followed by the sample synthesized at 150 °C without magnetic stirring (1:1), which shows a similar behavior.

The best electrical conductivity was obtained for the sample (1:1)s at 150 °C, followed by the sample (1:1)s at 100 °C.

These measurements give us an indication of how the pure NiMOF material behaves electrically, at 3 tons of pressure, which will give us an idea of what the performance of the electrochemical characterization will be like.

### 3.5. Results Electrochemical Characterization

Due to the high-purity phase, the set of samples (1:1)s was further utilized for electrochemical characterization, as described in the experimental section.

**Figure 7:**
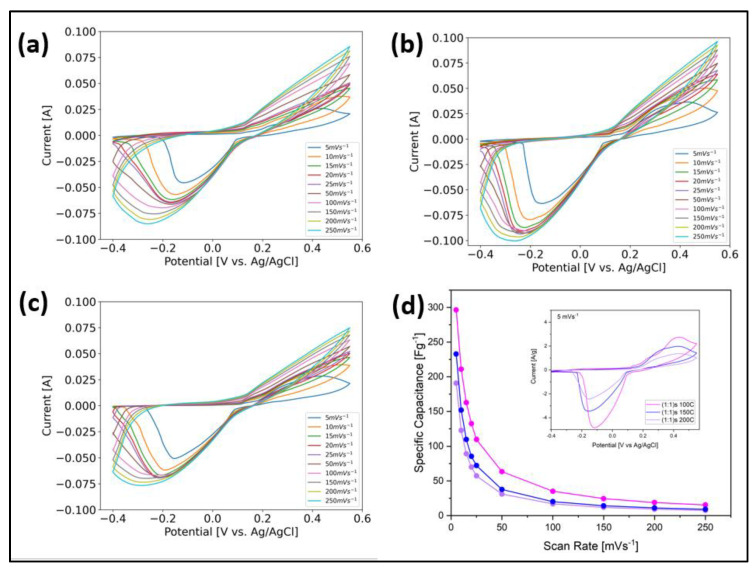
Cyclic voltammetry curve of the samples (**a**) (1:1)s 100 °C; (**b**) (1:1)s 150 °C; and (**c**) (1:1)s 200 °C. (**d**) Comparison between the different samples’ specific capacitances and CV curves at 5 mV/s.

The electrochemical performance was measured using a three-electrode configuration, using 2M KOH as an electrolyte for a potential window of [−0.4–0.55] V with a sweep rate from 5 mV/s to 250 mV/s. We used this wide potential window because, when the standard potential window was used (from 0.0 to 0.5 V), it was observed that the cyclic voltammetry curves intersected (Supplementary Information Figure S1), which implies that other chemical reactions were taking place due to the primary material, and probably that other phases formed (Fox and Akaba 1983). Also, Lee et al. reported that “Curve crossing stems from the increasing concentration of active catalyst—and so higher catalytic current—as the CV progresses” [31]. Figure 7 shows the cyclic voltammetry curves for the samples (a) (1:1)s 100 °C, (b) (1:1)s 150 °C, and (c) (1:1)s 200 °C, whose effective electrode masses were 9.42, 18.36, and 17.16 mg, respectively. For all three cases, it is observed that, as the scanning rate increases, the faradic contribution of oxidation–reduction of the sample is lost; in fact, it is observed that, from a scanning speed of 10 mV/s, the curves are observed to be smooth, without the characteristic peaks seen at 5 mV/s, which implies that at medium- and high-scanning speeds, the sample does not achieve charge exchange at the electrode–electrolyte interface.

The figures clearly show the pseudocapacitive behavior for the chosen potential window, whose faradic equations correspond to the equations:

$$NiO+OH^{-}↔NiOOH+e^{-}$$

$$Ni{\left(OH\right)}_{2}+OH^{-}↔NiOOH+H_{2}O+e^{-}$$

From the sample (1:1)s 100 °C Figure 7a, at a scan rate of 5 mV/s, it is observed that the maximum in the anodic peak gives rise to 0.43 V, while the cathodic peak occurs at −0.12 V, which corresponds to the reaction of $Ni{\left(OH\right)}_{2}$; a second anodic peak is also observed at 0.24 V and a convulsion for the cathodic peak that is broader due to the oxidation–reduction of NiO [32]. For the sample (1:1)s 150 °C (b), the maximum oxidation and reduction peaks occur at the voltages of 0.40 V and −0.12 V, respectively. Like the previous case, the contribution of the oxidation–reduction reaction of NiO is observed, in which an anodic shoulder is seen at around 0.32 V, along with an increase in the width of the cathodic peak determined by the formation of the $Ni{\left(OH\right)}_{2}$ phase. For the sample (1:1)s 200 °C, as shown in Figure 7c, the oxidation and reduction peaks occur at 0.44 V and −0.15 V, respectively, showing the contribution of the NiO species. When normalized by the effective mass, it is observed that, at 5 mV/s, the sample that has the greatest area is the (1:1)s 100 °C. From Figure 7d, the calculation of the specific capacitance according to Equation (1) is observed as a function of the scan rate. It is observed that, for a rate of 5 mV/s, the best electrochemical performance was obtained in the sample (1:1)s 100 °C with 296.46 F/g, followed by the sample (1:1)s 150 °C with 232.86 F/g, and finally the sample (1:1)s 200 °C with 190.74 F/g. These results agree with what is shown in Table 1, where it was expected that the electrochemical performance of the sample (1:1)s 200 °C, due to its greater electrical resistance, will be lower compared to those of other samples. Likewise, it is compared that, for the samples (1:1)s 100 °C and (1:1)s 150 °C, the sample synthesized at (1:1)s 100 °C has higher resistance and higher capacitance than the sample synthesized at 150 °C, while the latter has a lower resistance and lower capacitance; however, all three cases have the same order of magnitude. The synthesis temperature, ranging between 100 °C and 200 °C, did not significantly modify the crystalline structure of the NiMOFs; however, it did change their growth directions, their shapes, and their average particle sizes. The sample synthesized at 100 °C has the highest specific capacity and the sample at 200 °C has the lowest specific capacity.

**Figure 8:**
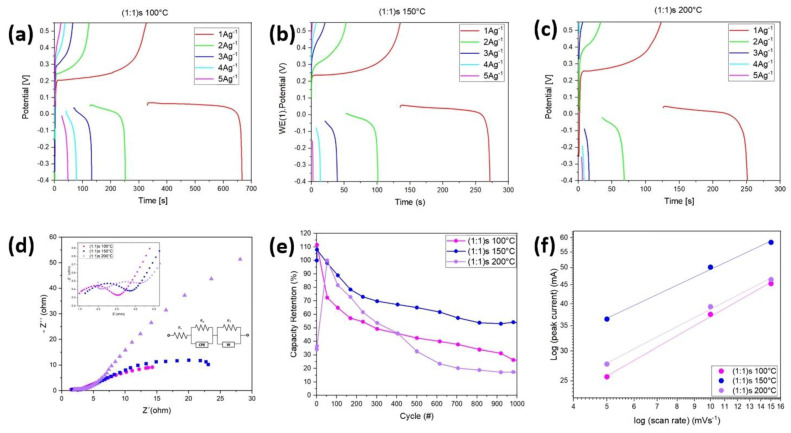
The galvanostatic charge and discharge performances at different currents for the samples (**a**) (1:1)s 100 °C; (**b**) (1:1)s 150 °C; and (**c**) (1:1)s 200 °C. (**d**) Nyquist plot of the three samples in the range of 0.1 MHz − 0.1 Hz and the Randles circuit; (**e**) capacity retention up to 1000 cycles; and (**f**) relationship between log (peak current) and log (scan rate) in the cathodic regions of the samples.

Figure 8 shows the galvanostatic charge and discharge (GCD) curve for samples (a) (1:1)s 100 °C, (b) (1:1)s 150 °C, and (c) (1:1)s 200 °C, for different currents of charge and discharge. The figures show the clear faradic behavior of the samples, where for small currents, the performance is low compared to higher charge and discharge currents. It is also observed that the sample (1:1)s 100 °C has a longer charge and discharge time, taking a total of 660 s for a current of 1 A/g, while the sample (1:1)s 150 °C takes around 270 s, and the sample (1:1)s 200 °C takes around 250 s. Using the equation to determine the specific capacitance, namely Equation (2), we have that, for the current densities of 1 A/g, 2 A/g, 3 A/g, 4 A/g, and 5 A/g, the specific capacitances for the sample (1:1)s 100 °C are 606.63 F/g, 487.02 F/g, 303.70 F/g, 348.97 F/g, and 277.84 F/g, respectively; for the sample (1:1)s 150 °C, the values are 307.33 F/g, 239.80 F/g, 155.21 F/g, 79.74 F/g, and 20.69 F/g, respectively; and for the sample (1:1)s 200 °C, the specific capacitances are 287.42 F/g, 127.74 F/g, 40.33 F/g, 32.80 F/g, and 33.92 F/g, respectively. In this way, the best performance is obtained for the sample (1:1) at 100 °C and, despite having a lower specific capacitance according to the cyclic voltammetry curves, it is through the charge–discharge curves that there is a more representative value by becoming independent of the amount of active material used to make the working electrode [33,34]. It is observed that the difference in voltage given between the charge and discharge is 0.50 V for the samples (1:1)s 100 °C and (1:1)s 150 °C, while for the sample (1:1)s 200°C, the sample showed a difference of 0.52 V. Thus, the resistance R_ESR_ can be determined, and for the samples (1:1)s 100 °C and (1:1)s 150 °C, a resistance ESR = 0.25 [Ω] is obtained, while for sample (1:1)s 200 °C, a resistance of ESR = 0.26 [Ω] is obtained, which is in accordance with what is observed in Table 1, given that the samples (1:1) synthesized at 100 and 150 °C have a lower internal resistance compared to the samples synthesized at 200 °C.

Figure 8d shows the Nyquist plot of the electrochemical impedance spectroscopy of the three samples in the range of 0.1 MHz–0.1 Hz, and the image shows that the samples (1:1)s 100 °C and (1:1)s 150 °C have the same behavior, while sample (1:1)s 200 °C has a rather inductive curve for the material. It is also observed that the samples (1:1)s 150 °C and (1:1)s 200 °C have a very high resistance to the diffuse layer. The vertical behavior of the slope is not observed in the Nyquist diagram at intermediate frequencies, so there is a limitation on the transport of ions between the electrolyte and the porous structure of the electrode, or the transport of ions is not uniform from the electrolyte. Since there is no dominant capacitive behavior of the electric double layer, and the polarization is due to a combination of kinetic and diffusion processes, and due to the shape of the impedance curves, the constant phase element (CPE) and Walburg impedance (W) elements are used. The circuit model of the three samples synthesized for a metal–ligand molar concentration of (1:1)s used stirring. Since we are interested in the representative capacitive element, the equivalent circuit is made for a frequency range where the semicircle is located. Thus, it is obtained that, for a range of 0.5 kHz–500 kHz, the simulation conditions for the sample (1:1)s 100 °C are $R_{s}=1.13\;\Omega$, $R_{p}=1.34\;\Omega$, $Y_{o}=240\;\mu s^{n}\Omega$, and n=0.68, $R_{1}=209\;\Omega$, and $\sigma =0.042\;\Omega \cdot s^{-1/2}$. In the range of 0.5 kHz–500 kHz, the simulation conditions for the sample (1:1)s 150 °C are $R_{s}=1.45\;\Omega$, $R_{p}=1.30\;\Omega$,$Y_{o}=149\;\mu s^{n}\Omega$, and n=0.73, $R_{1}=21.7\;\Omega$, and $\sigma =0.031\;\Omega \cdot s^{-1/2}$. And, in the range of 0.2 kHz–20 kHz, the simulation conditions for the sample (1:1)s 200 °C are $R_{s}=1.19\;\Omega$, $R_{p}=1.11\;\Omega$,$Y_{o}=300\;\mu s^{n}\Omega$, and n=0.75, $R_{1}=18.0\;\Omega$, and $\sigma =0.030\;\Omega \cdot s^{-1/2}$. This shows that it is the sample (1:1)s 150 °C that has the material with the highest internal resistance. The image in Figure 8f shows the capacitance retention of the samples (1:1)s 100 °C for a potential window of [−0.4–0.55] V. It is observed that, for 1000 cycles, the samples retain up to 30%, 54%, and 17% of the capacitance for the samples (1:1)s synthesized at 100 °C, 150 °C, and 200 °C, which is due to redox reactions occurring within the sample, causing the sample to decrease its specific capacitance. According to the CV diagram of the samples, we can study the energy storage mechanism of the NiMOFs, using the relationship between the peak current (J) and scan rate (v) according to the equations:

$$i=a\upsilon^{b}$$

$$Log\;\left(i\right)=\log\left(a\right)+b\log\left(v\right)$$

where *a* and *b* are adjustable parameters, *i* is the peak current density, and *v* is the scan rate. Thus, the *b* value of 1 suggests a capacitive-controlled process, while *b* = 0.5 indicates that the redox reactions are limited by a diffusion-controlled behavior [35]. Figure 8f shows that the b values of the cathode of NiMOFs are 0.52, 0.43, and 0.47 for the sample (1:1)s synthesized at 100 °C, 150 °C, and 200 °C, respectively, indicating the diffusion-controlled process.

From the synthesis of the NiMOF, using $NiCl_{2}$ as the salt and $H_{2}bdt$ as the ligand, at a molar concentration (metal:ligand) = (1:1)s, it was possible to optimize the synthesis, obtaining a homogeneous and uniform material, whose particles corresponded to nanostructured MOFs in the shape of rectangular prisms, and it was observed that a lower synthesis temperature is followed by a smaller particle size.

As can be seen from Figure 7 and Figure 8, and Table 1, the sample (1:1)s at 200 °C has the highest electrical and electrochemical resistance, showing a lower electrochemical performance compared to the samples synthesized at 100 °C and 150 °C. A similar behavior is observed when analyzing the capacitance, where, both electrically and electrochemically, the sample (1:1) at 100 °C has the highest capacitance, followed by the sample (1:1) at 150 °C, and finally the sample (1:1) at 200 °C. A direct relationship was not observed between the specific capacitance and the electrical conductivity; however, we had to consider that the sample (1:1)s 150 °C alone had a greater electrical conductivity than the sample (1:1)s 100 °C, while the sample (1:1)s 100 °C together with the binder and the activated carbon had a better electrochemical performance; that is, there was synergy in the performance of the cell and therefore greater ion exchange capacity between the interface and the KOH electrolyte, which was minor for the sample (1:1)s at 150 °C. However, the sample (1:1)s at 100 °C shows stability at 1000 cycles.

Table 2 shows the comparison between different electrochemically characterized Ni-based MOFs where, when comparing our nano NiMOF, it has a comparable specific capacitance, working in a broader potential window with a lower concentration of KOH electrolyte. Unlike in other studies, we obtained thinner nanorods and nanoflake-like structures, that is, 1D structures. Our best result was obtained at a synthesis temperature of 100 °C, which, when compared to the other works, requires a lower synthesis temperature to obtain the NiMOF, and does not exceed 130 °C, as has been performed and reported in previous studies [33,36,37]. Our synthesis is environmentally friendly, as compared to the reports mentioned in Table 2, where water is used as a solvent, while the others use solvents such as DMF, which makes the production of NiMOFs more expensive [33,38,39,40,41]. On the other hand, our samples were dried in vacuum at room temperature, without the need for temperature, whereas other syntheses require drying with temperature, so less energy is consumed [33,36,40]). Our synthesis is a one-step process, which makes it a direct method for obtaining nano NiMOFs [39] with high purity and high yield. Finally, we used this wide potential window because when the standard potential window was used (0.0–0.5 V). It was observed that the cyclic voltammetry curves intersect, which implies that other chemical reactions were taking place due to the primary material, namely, the creation of a new phase [31,42]. However, it is worth mentioning that two electrode measurements did not give a good response (Supplementary Information Figure S2), but many issues need to be addressed and resolved to make it a suitable electrode for two electrodes, such as adequate electrode substrate, suitable electrolyte, electrolyte concentration, and better sealing to avoid the evaporation of the electrolyte, etc.

## 4. Conclusions

A facile hydrothermal synthesis approach is suggested by the incorporation of the ultrasonication stage before putting the reactants in the furnace to achieve highly pure nickel-based nano MOF utilizing nickel chloride as a metal salt and H_2_bdt ligand at a molar concentration (1:1) and a synthesis temperature of 150 °C.

The rarely used H_2_bdt ligand was explored for NiMOF synthesis and the introduction of the ultrasonication stage led to the formation of a pure phase without impurities and free from oxides and the residues of the salt and ligands, and in high yield.

Other effective parameters such as synthesis temperature were also varied to 100 °C and 200 °C to see the effect on the final product. It was observed that, by increasing the synthesis temperature, the as-obtained NiMOF particles were more crystalline and displayed a larger size width. We had particles that were shaped like rectangular prisms with a particle width varying from 97.4 nm to 155.1 nm for (1:1)s 100 °C; from 94.9 nm to 292.28 nm for (1:1)s 150 °C; and from 187.0 nm to 349.8 nm for (1:1)s 200 °C. This increase in surface area due to the smaller size also indicated an increase in the specific capacitance at 100 Hz.

The electrochemical measurements revealed that all three samples synthesized using magnetic stirring exhibited a pseudocapacitive behavior, where the main redox reactions were Ni(OH)_2_ and NiO. From the cyclic voltammetry curves, the highest specific capacitance was obtained for the sample (1:1) at 100 °C, in agreement with the charge and discharge curves at different current densities, obtaining specific capacitance of 606.62 F/g, 307.33 F/g, and 287.42 F/g at a current density of 1 A/g for the samples synthesized at 100, 150, and 200 °C, respectively. Therefore, the synthesized nano NiMOF exhibited an improved specific capacitance.

It is worth mentioning that two electrode measurements did not give a very good response. Therefore, as a future aspect, many issues need to be addressed and resolved to make it a suitable electrode for two electrodes, such as an adequate electrode substrate, suitable electrolyte, electrolyte concentration, better sealing to avoid the evaporation of the electrolyte, etc.

## Figures and Tables

**Table 1:** The parallel capacitance, parallel resistance, and AC conductivity for the samples.

Sample	$C_{p100\mathrm{Hz}}$ [pF]	$R_{p100\mathrm{Hz}}$[MΩ]	$\sigma_{ac100\mathrm{Hz}}$ [µΩ cm^−1^]
(1:1)	17.553	155.359	0.102
(1:1)s 100°C	39.402	53.538	0.322
(1:1)s 150°C	27.739	40.559	0.484
(1:1)s 200°C	12.454	196.122	0.101

**Table 2:** Examples of nickel-based MOFs used as electrode material for supercapacitors.

MOF	$C_{s}$ [F/g]	Window Potential [V]	Electrolyte	Ref.
Hierarchical NiMOFs	1498.6	0.0–0.6	6M KOH	[33]
$\mathrm{NiMOF}\;(H_{2}bdc$)	804	0.0–0.6	2M KOH	[36]
NiMOF (BTC)	993	−0.1–0.5	6M KOH	[37]
Ni-based MOF nanorods	1698	0.0–0.6	6M KOH	[38]
NiMOF (PTA)	579	0.0–0.6	2M KOH	[39]
nano Ni-BPDC-MOF	488	0.0–0.6	3M KOH	[40]
$[Ni_{3}{\left(OH\right)}_{2}\left(C_{8}H_{4}O_{4}\right){\left(H_{2}O\right)}_{4}]\cdot 2H_{2}O$	988	0.0–0.6	3M KOH	[41]
NiMOF (1:1)s 100 °C	606.62	−0.4–0.55	2M KOH	This Work
NiMOF (1:1)s 150 °C	307.33			
NiMOF (1:1)s 200 °C	287.42			

## Data Availability

The original contributions presented in the study are included in the article and the supplementary material, further inquiries can be directed to the corresponding author.

## Supplementary Materials

The supporting information can be downloaded at: https://www.mdpi.com/article/10.3390/nano14040353/s1. Figure S1: CV curve at conventional potential window range. Figure S2: (a) Cyclic Voltammetry curve of the two-electrode configurations (b) Show the specific capacitance galvanostatic charge and discharge performance at different, (c) Nyquist plot for a range of 0.1 MHz to 0.01 Hz and Randles circuit, (d) and (e) capacity retention up to 5000 cycles, for the sample (1:1)s 100°C.

## References

[1] Yaghi O.M., Li H. (1995). Hydrothermal Synthesis of a Metal-Organic Framework Containing Large Rectangular Channels. J. Am. Chem. Soc..

[2] Taddei M., Petit C. (2021). Engineering metal–organic frameworks for adsorption-based gas separations: From process to atomic scale. Mol. Syst. Des. Eng. (R. Soc. Chem.).

[3] Bavykina A., Kolobov N., Il Son Khan J., Bau A., Gascon A., Ramirez J. (2020). Metal–Organic Frameworks in Heterogeneous Catalysis: Recent Progress, New Trends, and Future Perspectives. Chem. Rev..

[4] Yin H.-Q., Yin X.-B. (2020). Metal–Organic Frameworks with Multiple Luminescence Emissions: Designs and Applications. Acc. Chem. Res..

[5] Yang J., Yang Y. (2020). Metal–Organic Frameworks for Biomedical Applications. Small.

[6] Lawson H.D., Walton S.P., Chan C. (2021). Metal–Organic Frameworks for Drug Delivery: A Design Perspective. ACS Appl. Mater. Interfaces.

[7] Chi-Durán I., Enríquez J., Manquian C., Wrighton-Araneda K., Cañon-Mancisidor W., Venegas-Yazig D., Herrera F., Dinesh Pratap S. (2018). pH-Controlled Assembly of 3D and 2D Zinc-Based Metal-Organic Frameworks with Tetrazole Ligands. ACS Omega.

[8] Jia M., Xiong W., Yang Z., Cao J., Zhang Y., Xiang Y., Xu H., Song P., Xu Z. (2021). Metal-organic frameworks and their derivatives-modified photoelectrodes for photoelectrochemical applications. Coord. Chem. Rev..

[9] Calbo J., Golomb M.J., Walsh A. (2019). Redox-active metal–organic frameworks for energy conversion and storage. J. Mater. Chem. A (R. Soc. Chem. ).

[10] Raza H., Bai S., Cheng J., Majumder S., Zhu H., Liu Q., Chen G. (2023). Li-S Bateries: Challenges, Achievements and Opportunities. Electrochem. Energy Rev..

[11] Li C., Zhang L., Chen J., Li X., Sun J., Zhu J., Wang X., Fu Y.Y. (2021). Recent development and applications of electrical conductive MOFs. Nanoscale.

[12] Ma T., Li H., Ma J.-G., Cheng P. (2020). Application of MOF-based materials in electrochemical sensing. Dalton Trans..

[13] Raza H., Cheng J., Lin C., Majumder S., Zheng G., Chen G. (2023). High-entropy stabilized oxides derived via a low-temperature template route for high-performance lithium-surfur batteries. EcoMat.

[14] Sahoo S., Kumar R., Dhakal G., Shim J. (2023). Recent advances in synthesis of metal-organic frameworks (MOFs)-derived metal oxides and its composites for electrochemical energy storage applications. J. Energy Storage.

[15] Kuyuldar S., Genna D.T., Burda C. (2019). On the potential for nanoscale metal–organic frameworks for energy applications. J. Mater. Chem. A (R. Soc. Chem. ).

[16] Wang K.-B., Bi R., Wang Z.-K., Chu Y., Wu H. (2020). Metal–organic frameworks with different spatial dimensions for supercapacitors. New J. Chem. (R. Soc. Chem. ).

[17] Choi K.M., Jeong H.M., Park J.H., Zhang Y.-B., Kang J.K., Yaghi O.M. (2014). Supercapacitors of Nanocrystalline Metal–Organic Frameworks. ACS Nano.

[18] Sheberla D., Bachman J.C., Elias J.S., Sun C.-J., Shao-Horn Y., Dincă M. (2017). Conductive MOF electrodes for stable supercapacitors with high areal capacitance. Nat. Mater.

[19] Ouellette W., Darling K., Prosvirin A., Whitenack K., Dunbar K.R., Zubieta J. (2011). Syntheses, structural characterization and properties of transition metal complexes of 5,5′-(1,4-phenylene)bis(1H-tetrazole) (H_2_bdt), 5′,5′′-(1,1′-biphenyl)-4,4′-diylbis(1H-tetrazole) (H_2_dbdt) and 5,5′,5′′-(1,3,5-phenylene)tris(1H- tetrazole) (H_3_btt). Dalton Trans..

[20] Qiao C., Wei Q., Xia Z., Liang J., Chen S. (2011). Pb(II) and Mn(II) Coordination Compounds Involving 5,5′-(1,4-Phenylene)bis(1H-tetrazole):Synthesis, Characterization, and Effect on Thermal Decomposition of Ammonium Perchlorate. Chin. J. Chem..

[21] He X., An B.L., Li M.X. (2008). 5,5´-(p-Phenylene)di-1H-tetrazole. Acta Crystallogr. Sect. E.

[22] Kim B.K., Sy S., Yu A., Zhang J. (2015). Electrochemical Supercapacitors for Energy Storage and Conversion. Handbook of Clean Energy Systems.

[23] Vivas L., Jara A., Garcia-Garfido J.M., Serafini D., Singh D.P. (2022). Facile Synthesis and Optimization of CrOOH/rGO-Based Electrode Material for a Highly Efficient Supercapacitor Device. ACS Omega.

[24] Chand P., Sharma S. (2023). Supercapacitor and electrochemical techniques: A brief review. Results Chem..

[25] Larkin P. (2011). Infrared and Raman Spectroscopy, Principles and Spectral Interpretarion.

[26] Vanaraj R., Vinodh R., Periyasamy T., Madhappan S., Babu C.M., Asrafali S.P., Haldhar R., Raorane C.J., Hwang H., Kim H.-J. (2022). Capacitance Enhancement of Metal–Organic Framework (MOF) Materials by Their Morphology and Structural Formation. Energy Fuels.

[27] Kamath T.N., Vishnu P., Ramesh (2006). Synthesis of nickel hydroxide: Effect of precipitation conditions on phase selectivity and structural disorder. J. Power Sources.

[28] Cotton F.A., Wilkinson G. (1972). Advanced Inorganic Chemistry.

[29] Bonneviot L., Legendre O., Kermarec M., Daniéle O., Che M. (1990). Characterization by UV-vis-NIR reflectance spectroscopy of the exchange sites of nickel on silica. J. Colloid Interface Sci..

[30] Liu W., Migdisov A., Williams-Jones A. (2012). The stability of aqueous nickel(II) chloride complexes in hydrothermal solutions: Results of UV–Visible spectroscopic experiments. Geochim. Et Cosmochim. Acta.

[31] Lee K.J., McCarthy B.D., Dempsey J.L. (2019). On Decomposition, Degradation, and Voltammetric Deviation: The Electrochemist’s Field Guide to Identifying Precatalyst Transformation. Chem. Soc. Rev..

[32] Schiavi P.G., Altimari P., Marzolo F., Rubino A., Zanoni R., Pagnanelli F. (2021). Optimizing the structure of Ni–Ni(OH)2/NiO core-shell nanowire electrodes for application in pseudocapacitors: The influence of metallic core, Ni(OH)2/NiO ratio and nanowire length. J. Alloys Compd..

[33] Manikandan M.R., Cai K.P., Hu Y.D., Li C.L., Zheng Y.P., Liang Y.F., Song H.R., Shang M.Y., Shi X.N., Zhang J.X. (2021). Influence of hydrothermal reaction time on the supercapacitor performance of Ni-MOF nanostructures. Appl. Phys. A.

[34] Akin M., Zhou X. (2022). Recent advances in solid-state supercapacitors: From emerging materials to advanced applications. Int. J. Energy Res..

[35] Brousse T., Bélanger D., Long J.W. (2015). To Be or Not Be Pseudocapacitive?. J. Electrochem. Soc..

[36] Gao S., Sui Y., Wei F., Qi J., Meng Q., He Y. (2018). Facile synthesis of cuboid Ni-MOF for high-performance supercapacitors. J. Mater. Sci..

[37] Shi L., Yang W., Zha X., Zeng Q., Tu D., Li Y., Yang Y., Xu J., Chen F. (2022). In situ deposition of conducting polymer on metal organic frameworks for high performance hybrid supercapacitor electrode materials. J. Energy Storage.

[38] Xu J., Yang C., Xue Y., Wang C., Cao J., Chen Z. (2016). Facile synthesis of novel metal-organic nickel hydroxide nanorods for high performance supercapacitor. Electrochim. Acta.

[39] Jiang D., Wei C., Zhu Z., Xu X., Lu M., Wang G. (2021). Preparation of Flower-like Nickel-Based Bimetallic Organic Framework Electrodes for High-Efficiency Hybrid Supercapacitors. Crystals.

[40] Zhang W., Yin H., Yu Z., Jia X., Liang J., Li G., Li Y., Wang K. (2022). Facile Synthesis of 4,4´-biphenyl Dicarboxylic Acid-Based Nickel Metal Organic Frameworks with a Tunable Pore Size towards High-Performance Supercapacitors. Nanomaterials.

[41] Yan Y., Gu P., Zheng S., Zheng M., Pang H., Xue H. (2016). Facile synthesis of an accordion-like Ni-MOF superstructure for high-performance flexible supercapacitors. J. Mater. Chem. A.

[42] Fox M.A., Akaba R. (1983). Curve Crossing in the Cyclic Voltammetric Oxidation of 2-Phenylnorbornene. Evidence for an ECE Reaction Pathway. J. Am. Chem. Soc..

