# Supplementary material for: Synthesis and Optimization of Ni-Based Nano Metal–Organic Frameworks as a Superior Electrode Material for Supercapacitor
Source: Nanomaterials (Basel). 2024 Feb 13;14(4):353. doi: 10.3390/nano14040353 (PMC10892306; doi:10.3390/nano14040353)
Supplement: Supplementary file 1 [file nanomaterials-14-00353-s001.zip › nanomaterials-2804695-supplementary.pdf]

Supplementary Information (SI -1)

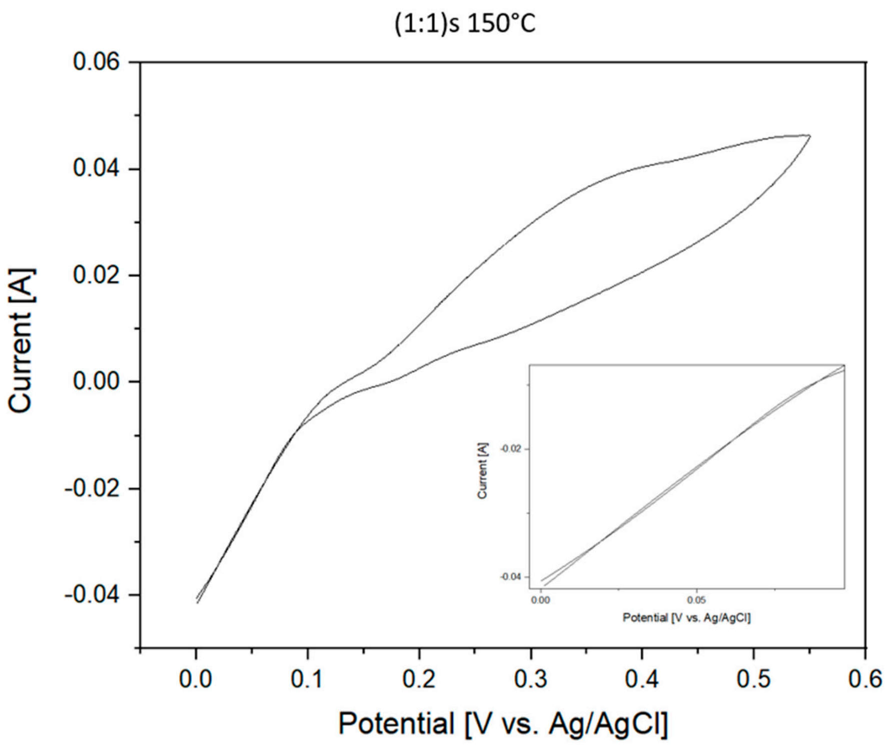

Figure S1: CV curve at conventional potential window range.

Supplementary Information (SI -2)

The Standard two-electrode Configuration
The configuration used for the two-electrodes configuration was the manufacture of a coin cell. The same mixture of the working electrode was deposited on thin stainless-steel discs, these were left on the plate until the solvent had evaporated. The electrodes were separated using a filter paper moistened with 2M KOH. The coin cell is compacted with a stainless-steel cell at a pressure of 1.33 MPa.

The measurements of the electrochemical properties that were studied for this electrode configuration were cyclic voltammetry (CV) with scan rates of 5 mVs^{-1} to 250 mVs^{-1}, Galvanostatic impedance measurements over a frequency range of 0.01 MHz to 0.1 Hz, and Charge-Discharge and cyclability performance was made in a potential window of [-0.4 to 0.55] V, for a current density of 0.01, 0.02 and 0.03 Ag^{-1}, and 0.01 Ag^{-1} respectively.

Results.
Figure (a) show the CV curve for the symmetric coin cell, no redox peaks are produced or observed in the cyclic voltammetry curve, showing low area. When calculating the specific capacitance, we obtained at 5mV/s, 1.2 F/g, figure (b).
(c) The Nyquist diagram shows that at high frequencies, the three samples have a large arc semicircle with a small line at low frequencies, the simulation conditions for the Randles circuit are $R_s = 3.81\ \Omega$, $R_p = 116.20\ \Omega$,$Y_o = 2.90\ m\ s^n\Omega$, and $n = 0.97$, and $\sigma = 0.099\ \Omega \cdot s^{-1/2}$, showing a high internal resistance.
(d) The curve of galvanostatic charge-discharge shows pseudocapacitance performance, where we obtained a specific capacitance of 0.15 F/g at 0.01 A/g.
Finally, the cyclability, figure (e), show a retention up the 40% for 5000 cycles at 0.01A/g, showing a degradation of the material.

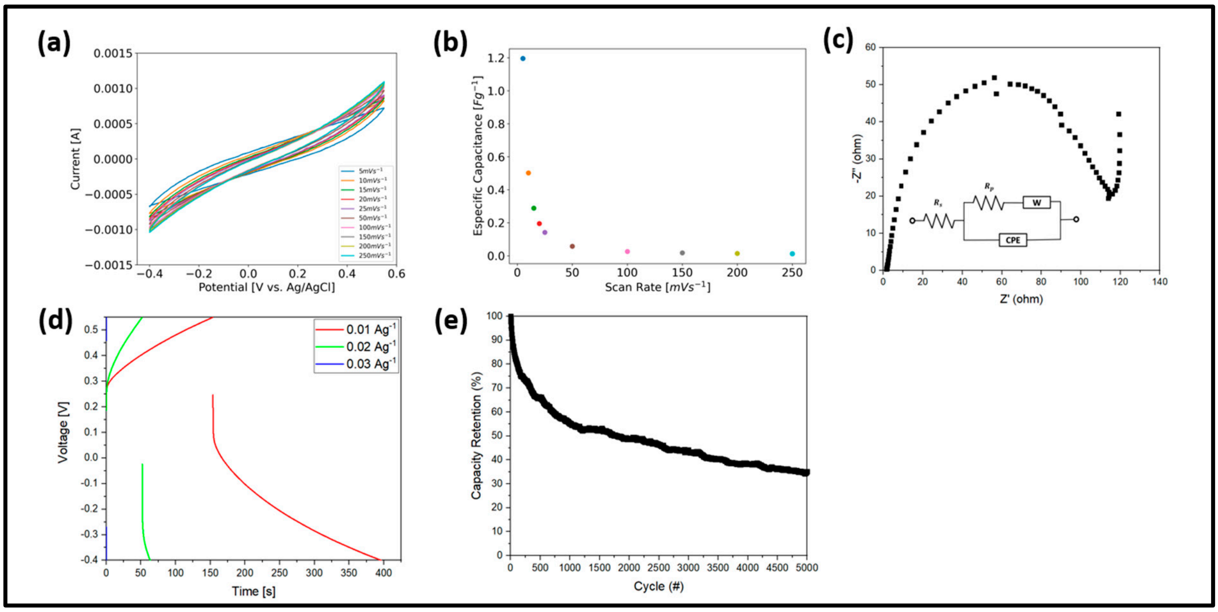

Figure S2. Shows the (**a**) Cyclic Voltammetry curve of the two-electrode configurations (**b**) Show the specific capacitance galvanostatic charge and discharge performance at different, (**c**) Nyquist plot for a range of 0.1 MHz to 0.01 Hz and Randles circuit, (**d**) and (**e**) capacity retention up to 5000 cycles, for the sample (1:1)s 100°C.
